# Digestive symptoms of COVID-19 and expression of ACE2 in digestive tract organs

**DOI:** 10.1038/s41420-020-00307-w

**Published:** 2020-08-11

**Authors:** Jiabin Xu, Mei Chu, Fan Zhong, Xinghua Tan, Guofang Tang, Jianbo Mai, Niangmei Lai, Chenyu Guan, Yujie Liang, Guiqing Liao

**Affiliations:** 1 Department of Oral and Maxillofacial Surgery, Hospital of Stomatology, Sun Yat-sen University, 56th Lingyuanxi Road, 510055 Guangzhou, Guangdong China; 2 Guangdong Province Key Laboratory of Stomatology, No. 74, 2nd Zhongshan Road, 510080 Guangzhou, Guangdong China; 3 Guanghua School of Stomatology, Sun Yat-sen University, 56th Lingyuanxi Road, 510055 Guangzhou, Guangdong China; 4 Guangzhou Eighth People’s Hospital, Guangzhou Medical University, 510060 Guangzhou, Guangdong China; 5 School of Stomatology, Wuhan University, 237th Luoyu Road, 430079 Wuhan, Hubei China

**Keywords:** Transcription, Infectious diseases

## Abstract

SARS-CoV-2 has resulted in numerous cases of Coronavirus Disease 2019 (COVID-19) worldwide. In addition to fever and respiratory symptoms, digestive symptoms also are observed in some patients with COVID-19. Angiotensin-converting enzyme 2 (ACE2) was reported to be the receptor for SARS-CoV-2. The aim of this study was to comprehensively investigate the digestive symptoms that occur in COVID-19 patients, and the potential pathogenic route of the SARS-CoV-2 infection in digestive tract organs (from the oral cavity to the gastrointestinal tract). We investigated the digestive symptoms of 48 patients with COVID-19 and explored ACE2 expression in digestive tract and lung cancers, based on a series of bulk and single-cell RNA sequencing data obtained from public databases. We found that 25% (12/48) of the patients with COVID-19 suffered from digestive symptoms, among which pharyngalgia (7/48) was the most common manifestation, followed by diarrhea (3/48), anorexia (3/48), and nausea (1/48). The bulk tissue RNA sequencing analysis indicated that digestive tract organs had higher ACE2 expression levels compared to the lung, and the expression of ACE2 in the lung increased with age. Single-cell RNA-Seq results showed that the ACE2-positive-cell ratio in digestive tract organs was significantly higher compared to the lung. ACE2 expression was higher in tumor cells compared to normal control (NC) tissues. While in gastric tissues, ACE2 expression gradually increased from chronic gastritis to metaplasia, to early cancer. Our data might provide a theoretical basis for screening the SARS-CoV-2 susceptible population and for the clinical classification of treatment of patients with COVID-19.

## Introduction

The global pandemic, Coronavirus Disease 2019 (COVID-19), caused by Severe Acute Respiratory Syndrome Coronavirus 2 (SARS-CoV-2) infection^1^, has been raging throughout the world. By June 25, 2020, COVID-19 has been found in 215 countries, with a total of >9.2 million confirmed cases worldwide, including over 479,000 deaths^2^.

It has been established that SARS-CoV-2 invades host cells by binding to the transmembrane receptor Angiotensin-converting enzyme 2 (ACE2)^3^, which also is the receptor for SARS-CoV and HCoV-NL63^4,5^. ACE2 plays a crucial role in the cellular entry for SARS-CoV-2, which means that ACE2-positive cells may act as target cells and are susceptible to infection^6^. Thus, the expression of ACE2 might affect the invasion path and pathogenicity of the virus.

The common symptoms of COVID-19 include fever, fatigue, cough, myalgia, and dyspnea^7–9^. Also, some patients may suffer from digestive system symptoms, such as pharyngalgia, diarrhea, nausea, vomiting, and abdominal pain^10^, suggesting that the digestive tract organs also may be targeted by the virus. Studies have reported that ACE2 was expressed in lung, esophagus epithelial cells, ileum^11^, colon^12^, kidney, bladder^13^, and oral mucosa^14^. However, information is lacking concerning the comparative analysis of ACE2 expression in the entire digestive tract from the oral cavity through the gastrointestinal tract. The oropharynx and gastrointestinal tract are physiologically interlinked and share similar microbial environments^15^. They also share similarities with respect to the microenvironment associated with epithelial carcinogenesis. Reports show that oral bacteria play a role in the genesis of digestive tract tumors^16–18^. More than 1% of COVID-19 cases co-exist with malignant tumors^10^. It is unclear whether there are differences in ACE2 expression between tumor and normal cells.

In this study, we assessed the clinical characteristics and investigated the digestive symptoms of 48 patients with COVID-19 in Guangzhou Eighth People’s Hospital. We explored ACE2 expression in digestive tract cancers and lung cancers, based on a series of bulk tissue RNA sequencing data from two independent databases, and single-cell transcriptome data from three single-cell databases. The results showed that nearly a quarter of COVID-19 patients had gastrointestinal symptoms, that ACE2 expression was higher in the digestive tract than the lung, and the ratio of ACE2-positive cells in tumor tissues was higher than that in paracancerous normal tissue.

## Materials and methods

### Patients’ involvement and data collection

Forty-eight hospitalized patients (admitted from January 26 to March 20, 2020) in Guangzhou Eighth People’s Hospital (the hospital exclusively designated for COVID-19 patients in Guangzhou), who were clinically diagnosed with “viral pneumonia” based on their clinical symptoms (fever or respiratory symptoms) with typical changes in chest radiology, were preliminarily included in this study. Patients without or with negative test results were excluded from this study.

Demographic information, clinical characteristics (including medical history, comorbidities, signs, and symptoms) were obtained from the electronic medical record system of Guangzhou Eighth People’s Hospital and analyzed by three independent researchers. All patients included in our study provided consent for the nasopharyngeal swabs and clinical information collection. This study was approved by the institutional ethics board of Guangzhou Eighth People’s Hospital (No. 202006139) and complied with the Declaration of Helsinki concerning medical research using human subjects.

### Nucleic acid detection of SARS-CoV-2

Nasopharyngeal swabs were collected and placed into a sterile tube containing RNA preservation solution. The swabs were sent for SARS-CoV-2 RNA extraction and detection within 1 h by a real-time reverse transcriptional polymerase chain reaction (RT-PCR) system by following the commercial test kit instructions (Da’an Gene cooperation, Cat DA0930) as previously described^19^. Briefly, two PCR primer and probe sets targeting ORF1a/b and nCoV-N genes were separately added into the same reaction tube. Positive and negative controls were involved for detection.

### Bulk tissue RNA sequencing (RNA-Seq) data analysis

The RNA-seq data (level 3) and clinical information for samples were downloaded from The Cancer Genome Atlas (TCGA) (https://portal.gdc.cancer.gov/repository) using the GDC Data Transfer Tool. The datasets used for ACE2 analysis included head and neck squamous cell carcinoma (HNSC), esophageal carcinoma (ESCA), stomach adenocarcinoma (STAD), colon adenocarcinoma (COAD), lung adenocarcinoma (LUAD), and lung squamous cell carcinoma (LUSC). Gene expression was presented as log_2_ (Fragments Per Kilobase per Million mapped reads (FPKM) + 1). The effects of age and sex on ACE2 expression were explored. We also downloaded the boxplot for ACE2 expression in human cancer cell lines from the Cancer Cell Line Encyclopedia (CCLE) (https://portals.broadinstitute.org/ccle) database for verification.

### Single-cell RNA sequencing (scRNA-Seq) data analysis

The lung cancer dataset, EXP0068, downloaded from the Cancer Single-cell State Atlas (http://biocc.hrbmu.edu.cn/CancerSEA/home.jsp), and the lung tissue dataset, SRA878024, downloaded from the PanglaoDB (https://panglaodb.se/index.html), were merged for further analysis. The esophageal squamous cell carcinoma (ESCC) dataset GSE81812, oral cancer dataset GSE103322, gastric cancer dataset GSE134520, and colorectal cancer dataset GSE81861 were obtained from the Gene Expression Omnibus (GEO) (https://www.ncbi.nlm.nih.gov/geo/). For GSE103322, cells from lymph nodes were removed. The R package Seurat version 3.1.1^20^ was used for single-cell data analysis. Gene-by-barcode count matrices were normalized, log-transformed, and scaled, followed by dimension reduction using principal components analysis (PCA). Uniform manifold approximation and projection (UMAP) was used to carry out dimensionality reduction and clustering.

All analyses were performed in R (R version 3.6.1) and GraphPad 8, and the level of significance was set at *p* ≤ 0.05.

## Results

### Demographics and clinical characteristics

A total of 48 patients diagnosed as COVID-19 were included in the study. Twenty-five patients were male, and 23 were female. The median patient age was 41 years and ranged from 18 to 90 years. The majority (75%) of the patients included in the study were under 60 years of age. 27% (13/48) of the patients had at least one underlying comorbidity, the most common of which was hypertension (9/48). Three patients suffered from diabetes, chronic obstructive pulmonary disease (COPD), or chronic renal diseases, respectively. Two patients presented with cardiovascular and cerebrovascular disease. One patient had chronic liver disease, malignancy, or digestive system disease, respectively. The most common symptoms were fever or cough (29/48), followed by digestive symptoms (12/48), fatigue (6/48), or dyspnea (2/48). Among patients who presented with digestive system symptoms, pharyngalgia (7/48) was the most common manifestation, followed by diarrhea (3/48), anorexia (3/48), and nausea (1/48) (Table 1).

**Table 1:** Demographics and clinical characteristics of patients with COVID-19.

Total number of cases, no.	48
Age, year-median(range)	41 (18–90)
Age group, no.	
<60 years	36
≥60 years	12
Sex, no.	
Male	25
Female	23
Comorbidity, no.	13
Hypertension	9
Diabetes	3
Chronic obstructive pulmonary disease (COPD)	3
Chronic renal diseases	3
Cardiovascular and cerebrovascular diseases	2
Chronic liver diseases	1
Malignancy	1
Digestive system disease	1
Signs and symptoms, no.	
Fever	29
Cough	29
Digestive symptoms	12
Pharyngalgia	7
Diarrhea	3
Anorexia	3
Nausea	1
Fatigue	6
Dyspnea	2

### Bulk tissue RNA-Seq data analysis

**Fig. 1:**
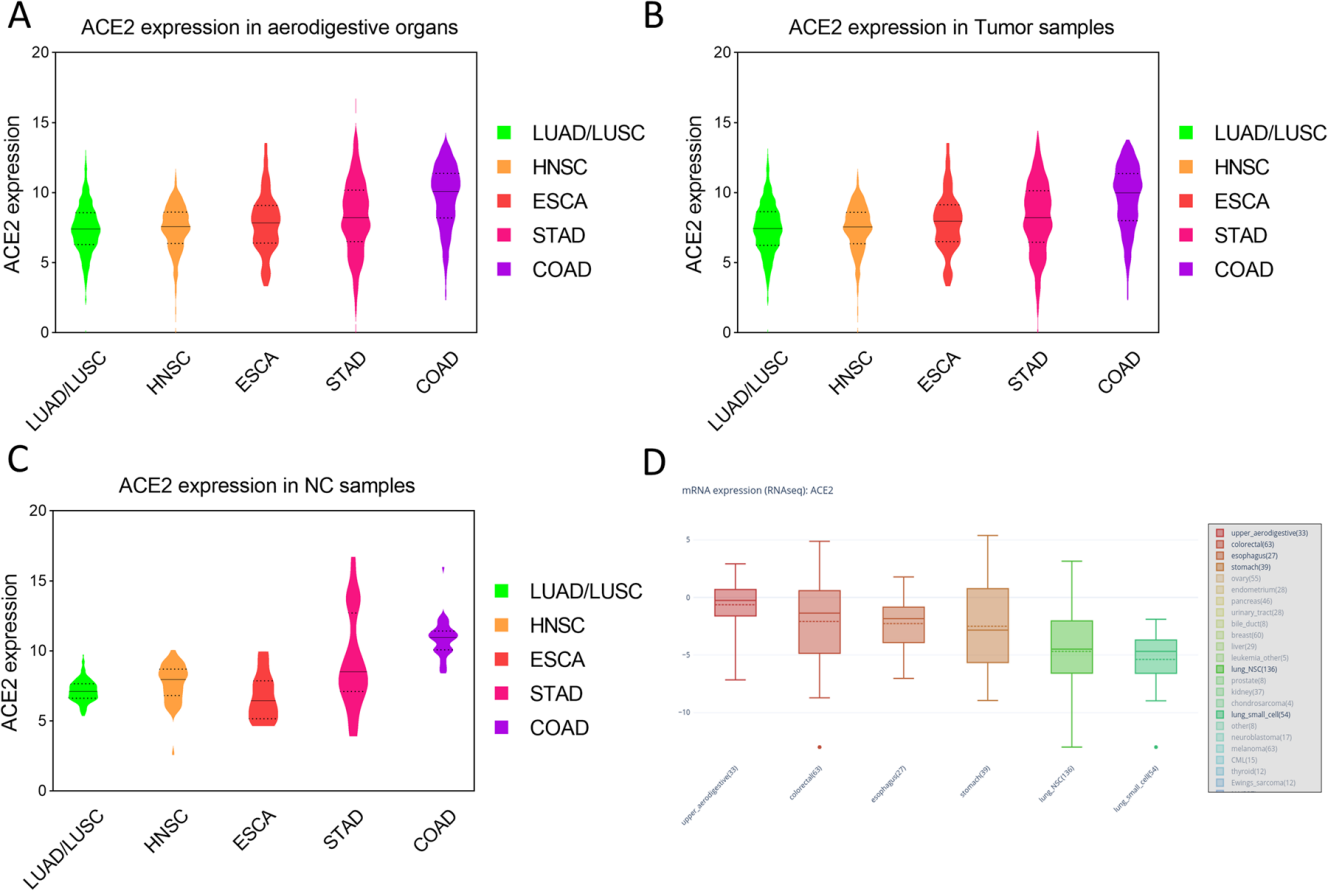
The expression of ACE2 in different tissues. **a**–**c** ACE2 expression of different tissues, downloaded from TCGA database. **a** ACE2 expression in all samples; **b** ACE2 expression in tumor samples; **c** ACE2 expression in NC samples. Black solid line, median value; black dotted line, interquartile range. **d** ACE2 expression in cancer cell lines, downloaded from CCLE database. Solid line, mean value; dotted line, median value. ACE2 Angiotensin-converting enzyme 2, TCGA The Cancer Genome Atlas, NC healthy normal control, LUAD lung adenocarcinoma, LUSC lung squamous cell carcinoma, HNSC head and neck squamous cell carcinoma, ESCA esophageal carcinoma, STAD stomach adenocarcinoma, COAD colon adenocarcinoma, CCLE Cancer Cell Line Encyclopedia.

A total of 2556 tumor samples and 236 normal control (NC) samples were downloaded from the TCGA database. Table 2 shows the expression of ACE2 (log_2_(FPKM + 1)) in the different samples. Figure 1a–c, respectively, show ACE2 expression in all tumor and NC samples from the different tissues. The results showed that digestive tract organs had higher ACE2 expression compared to the lung. In digestive tract cancers, ACE2 expression gradually increased from the oral cavity to the esophagus, stomach, and the colon, following the path of the digestive tract (Fig.1a–c). The results were validated using the cancer cell line data from the CCLE dataset (Fig. 1d).

**Table 2:** ACE2 expression in aerodigestive cancers.

Cancer	Sample type	Sample size No.	Mean expression	Standard deviation
			log_2_ (FPKM + 1)	
Lung	NC	108	7.174	0.8162
	Tumor	1037	7.446	1.937
Oral	NC	44	7.714	1.406
	Tumor	502	7.363	1.843
Esophagus	NC	11	6.777	1.716
	Tumor	162	7.91	2.186
Stomach	NC	32	9.324	3.447
	Tumor	375	8.18	2.633
Colon	NC	41	10.85	1.326
	Tumor	480	9.574	2.354

Note: data was downloaded from The Cancer Genome Atlas (TCGA).

*ACE2* angiotensin-converting enzyme 2; *NC* normal control*, ACE2* angiotensin-converting enzyme 2; *NC* normal control.

**Fig. 2:**
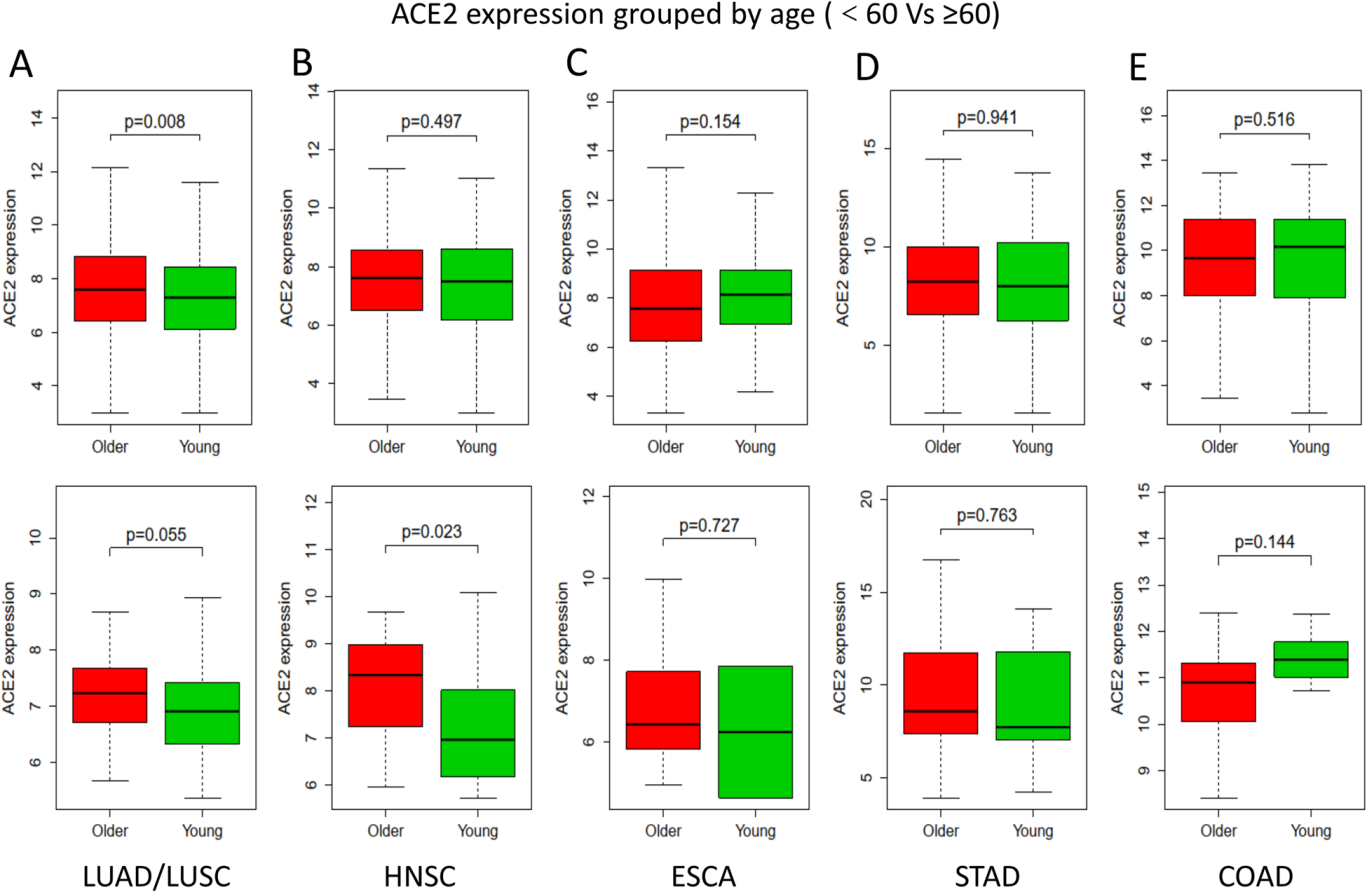
The expression of ACE2 in different organs grouped by age. **a**–**e** The expression of ACE2 in different tissues downloaded from TCGA database, grouped by age. Top row, the expression of ACE2 in tumor samples; Bottom row, the expression of ACE2 in NC samples. Bold black line, median value; dotted black line, range of values. Statistical tests: Mann–Whitney *U-*test. ACE2 angiotensin-converting enzyme 2, TCGA The Cancer Genome Atlas, NC healthy normal control, LUAD lung adenocarcinoma, LUSC lung squamous cell carcinoma, HNSC head and neck squamous cell carcinoma, ESCA esophageal carcinoma, STAD stomach adenocarcinoma, COAD colon adenocarcinoma.

**Fig. 3:**
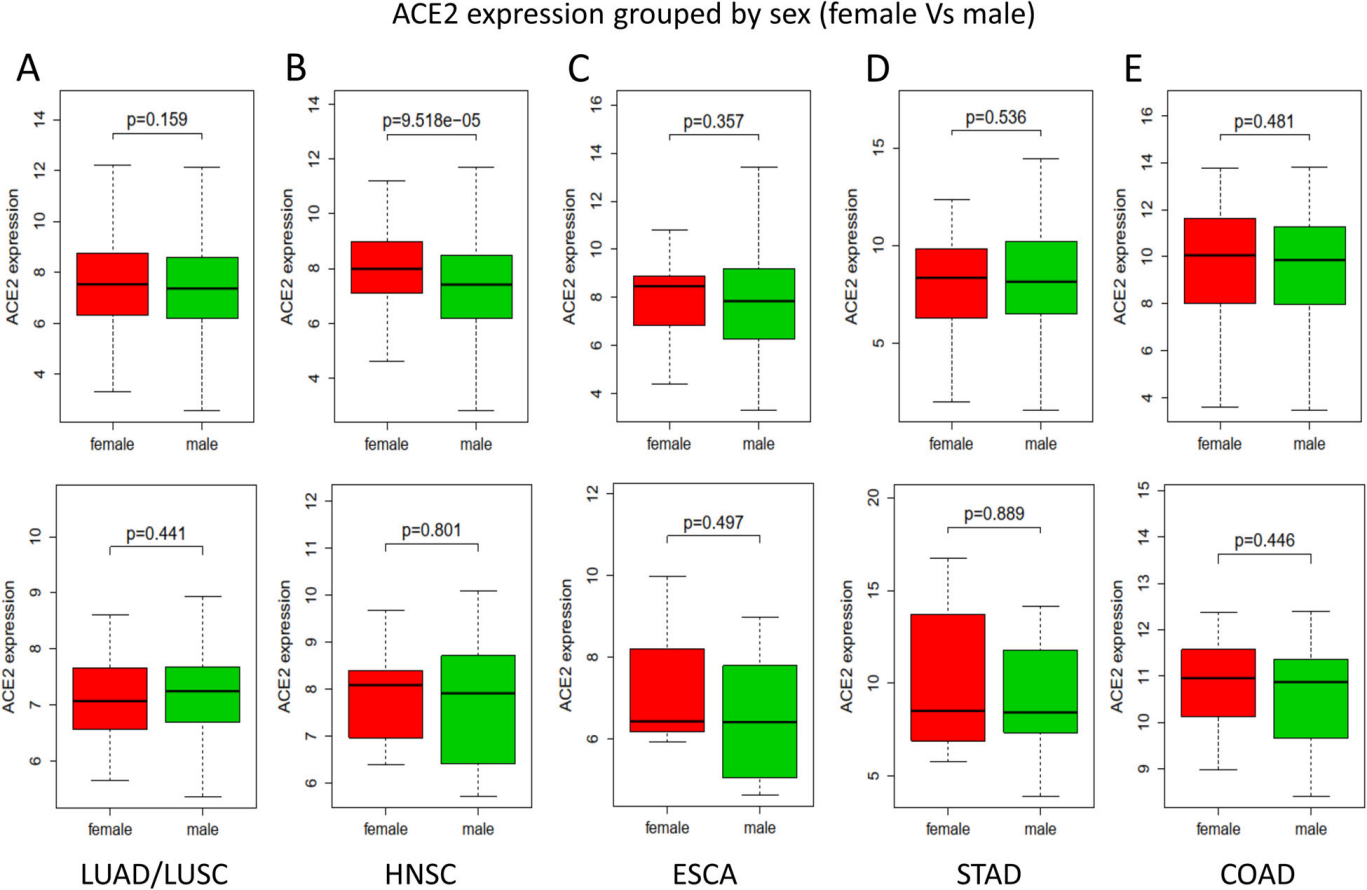
The expression of ACE2 in different organs grouped by sex. **a**–**e** The expression of ACE2 in different tissues downloaded from TCGA database, grouped by sex. Top row, the expression of ACE2 in tumor samples; Bottom row, the expression of ACE2 in NC samples. Bold black line, median value; dotted black line, range of values. Statistical tests: Mann–Whitney *U-*test. ACE2 angiotensin-converting enzyme 2, TCGA The Cancer Genome Atlas, NC healthy normal control, LUAD lung adenocarcinoma, LUSC lung squamous cell carcinoma, HNSC head and neck squamous cell carcinoma, ESCA esophageal carcinoma, STAD stomach adenocarcinoma, COAD colon adenocarcinoma.

To analyze the effect of age or sex on ACE2 expression, the patients were divided into a young group (<60) and an older group (≥60) based on age (Fig. 2), and the patients also were separated into females and males (Fig. 3), based on the clinical data in the TCGA database. The results revealed that for lung cancer, based on either tumor samples or NC samples (Fig. 2a), ACE2 expression in the older group was higher compared to the young group. Also, for oral cancer, the expression of ACE2 was significantly increased in the older group for the NC samples (Fig. 2b), as well as in the female group for the tumor samples (Fig. 3b).

### ScRNA-Seq data analysis

**Fig. 4:**
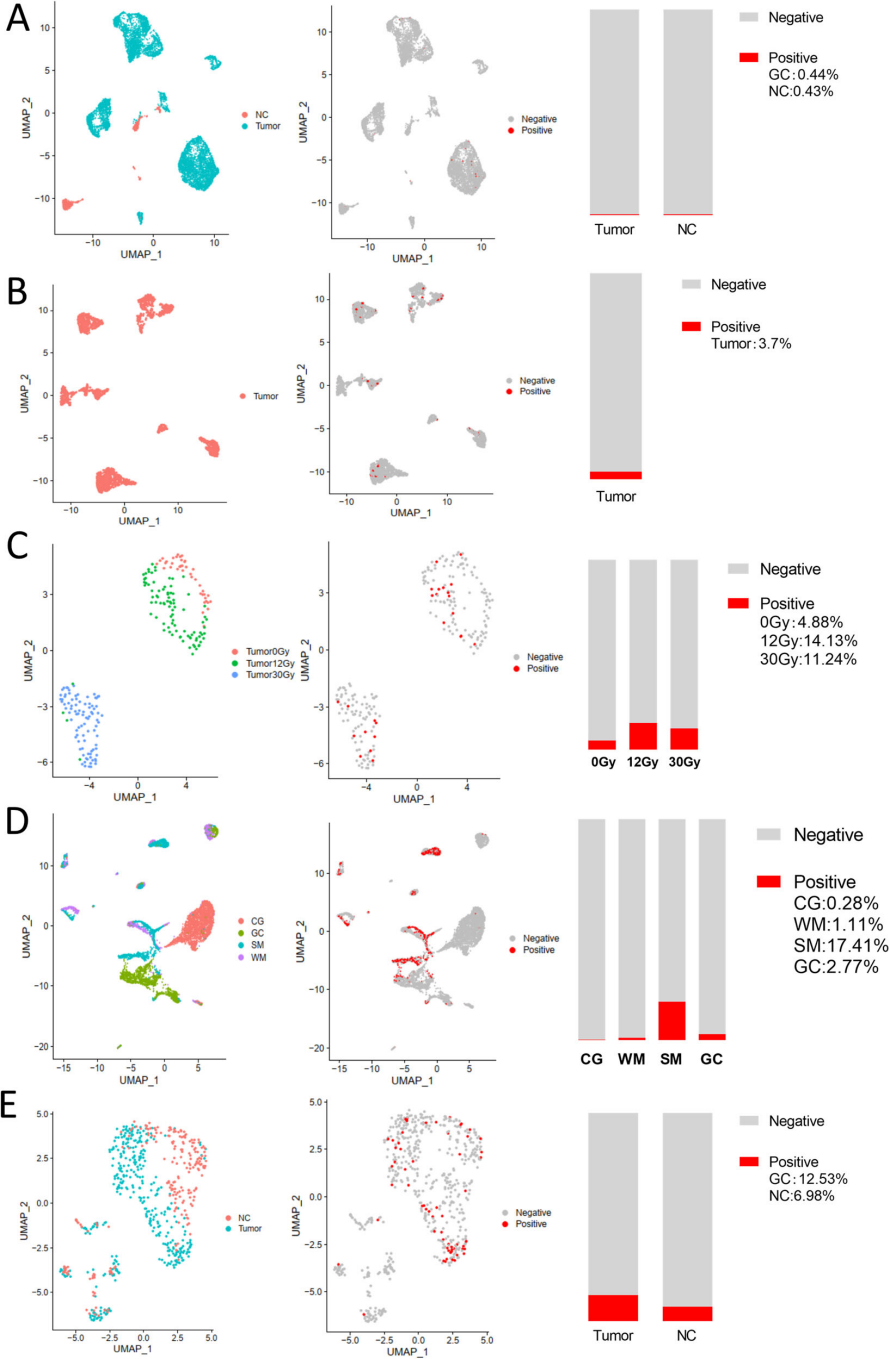
Single-cell sequensing analysis of aerodigestive cancer cells. **a** Single-cell analysis of lung cancer cells (tumor) in EXP0068 and NC cells in SRA878024. **b** Single-cell analysis of oral cancer cells in GSE103322. **c** Single-cell analysis of ESCC cell line KYSE-180 (tumor) treated with different doses of radiotherapy in GSE81812. **d** Single-cell analysis of CG, WM, SM, and GC cells in GSE134520. **e** Single-cell analysis of colon cancer cells (tumor) and NC cells in GSE81861. Left column, UMAP plots showing the distribution of cells, color-coded for pathology; middle column, UMAP plots showing the distribution of ACE2-positive cell (red); right column, Stacked barplot showing the proportion of ACE2-positive cells (red). EXP0068 was downloaded from the Cancer Single-cell State Atlas; SRA878024 was downloaded from the PanglaoDB; GSE103322, GSE81812, GSE134520, and GSE81861 were obtained from the Gene Expression Omnibus. NC healthy normal control, ESCC esophageal squamous cell carcinoma, CG chronic gastritis, WM wild intestinal metaplasia, SM severe intestinal metaplasia, GC gastric cancer, UMAP Uniform Manifold Approximation and Projection, ACE2 angiotensin-converting enzyme 2.

**Fig. 5:**
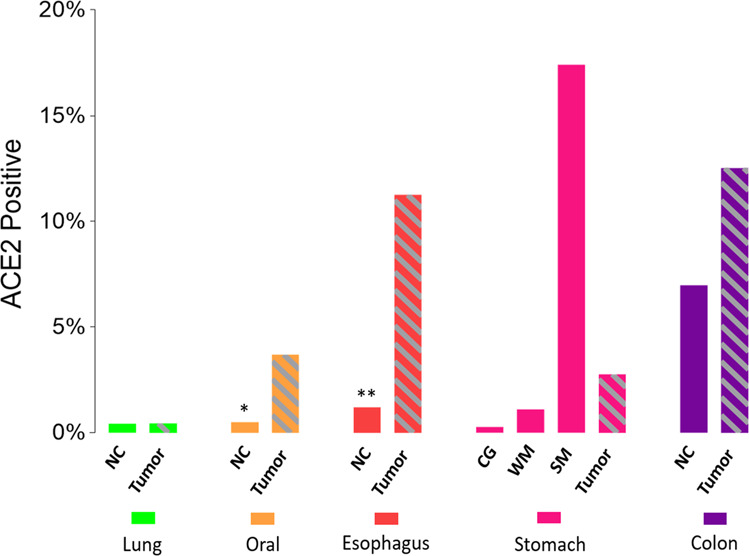
ACE2 expression increases along with the path of digestive tract. ACE2-positive cell proportion in aerodigestive cancers. *ACE2-positive cell proportion in oral mucosa reported by Xu et al.^14^; **ACE2-positive cell proportion in esophageal epithelium reported by Zou et al.^11^. ACE2 angiotensin-converting enzyme 2, NC healthy normal control, CG chronic gastritis, WM wild intestinal metaplasia, SM severe intestinal metaplasia, GC gastric cancer.

Six different scRNA-Seq datasets were included in this study. SRA878024 and EXP0068 were lung normal tissue or lung cancer scRNA-Seq data, respectively. GSE103322 was oral cancer scRNA-Seq data. GSE81812 was scRNA-Seq data for the ESCC cell line, KYSE-180, treated with different doses of radiotherapy. GSE134520 included scRNA-Seq data from chronic gastritis (CG), wild intestinal metaplasia (WM), severe intestinal metaplasia (SM), and gastric cancer (GC). GSE81861 included scRNA-Seq data from colon cancer and adjacent normal tissues. The results showed that ACE2-positive cells accounted for the lowest proportion in lung samples, with 0.43% (3/705) in the NC group and 0.44% (33/7447) in the tumor group (Fig. 4a). The ACE2-positive rate in oral cancer cells was 3.7% (75/2027) (Fig. 4b), while the ACE2-positive ratio in KYSE-180 treated with different doses of radiotherapy was, 4.88% (2/41) for 0 Gy, 14.13%(13/92) for 12 Gy, and 11.24% (10/89) for 30 Gy (Fig. 4c). The ratios of ACE2-positive cells in the CG, WM, SM, and GC groups were 0.28% (83/29678), 1.11% (59/5325), 17.41 (3017/14310), and 2.77% (114/4110), respectively (Fig. 4d). The ACE2-positive-cell ratio was 6.98% (15/215) in normal colonic mucosal cells, and 12.53% (47/375) in colon cancer (Fig. 4e). The ACE2-positive rate in the esophageal and oral mucosa was reported to be nearly 1.2%^11^ and 0.52%^14^, respectively. We found that the ACE2-positive-cell ratio in digestive tract organs was significantly higher than in the lung. The ACE2 expression also was higher in tumor cells compared to NC tissues. The ACE2 expression in gastric tissues gradually increased from chronic gastritis to metaplasia, then cancer (Fig. 5).

## Discussion

This study investigated the gastrointestinal symptoms of 48 patients with COVID-19 who were admitted to the Guangzhou Eighth People’s Hospital. We explored ACE2 expression in digestive tract cancers and lung cancers, based on both bulk tissue RNA-Seq data and scRNA-Seq data.

The median age of the 48 patients was 41 years, which was similar to data reported by Xu et al. (41 years)^21^, but was younger than the ages found in many other reports^8,9,22,23^. Consistent with other recent reports, hypertension and diabetes were the most common underlying comorbidities, and fever and cough were the most common symptoms of COVID-19 infection^9,23^. In this study, 25% of the COVID-19 patients exhibited digestive symptoms, which was lower than the percentage reported by Xu et al.^21^ and Zhang et al.^22^. The reason for this difference may be that the patients in this study were younger and presented with fewer underlying digestive system diseases. The most common digestive symptom was pharyngalgia, which was consistent with other studies^22,24^.

We observed that the expression of ACE2 in the lung increased with age but was independent of sex, which was consistent with the report by Chen et al.^25^. This may partly explain why older patients with COVID-19 are more likely to develop pneumonia.

This study found that ACE2 was highly expressed in digestive tract tumors or paracancerous tissues, compared to the lung for both the bulk tissue analysis and the single-cell analysis. Elevated ACE2 expression in the digestive tract suggested that the digestive tract organs also should be considered to be vulnerable targets for SARS-CoV-2 infection. It was reported that SARS-CoV-2 might cause a cytokine storm and multi-organ failure in severe pneumonia patients^26^. Similarly, SARS-CoV-2 might interact with ACE2 in digestive organs, causing further damage to the mucous membrane barrier, and increase inflammatory cytokine production. Oral-related symptoms are rarely reported with COVID-19 infections, but this does not indicate that an oral infection route for SARS-CoV-2 should be excluded. The oral cavity and gastrointestinal organs share similarities in the microbiome, inflammation, and tumorigenesis. Thus, we expect that the oral and gastrointestinal organs should exhibit common interactions in the route of SARS-CoV-2 infection. Furthermore, recent studies have reported a high positive rate of SARS-CoV-2 detection in saliva^27,28^, indicating that oral infection could be an early symptom of COVID-19.

Interestingly, we found that the ACE2-positive rate in tumor cells was significantly higher compared to the NC group. Moreover, ACE2 expression in gastric tissues gradually increased from chronic gastritis to intestinal metaplasia, to early gastric cancer. Similar findings were reported for the colon^29^. These results suggested that cancer patients might have a higher risk of SARS-CoV-2 infection. From another perspective, we suspected that increased expression levels of ACE2 affected the occurrence of digestive tract tumors. ACE2 hydrolyzes Ang II to Ang-(1–7), which negatively regulates the active Renin-Angiotensin System (RAS). Reports show that Ang-(1–7) plays an inhibitory role in the genesis and development of tumors^30,31^. However, SARS-CoV-2 could cause an inflammatory cytokine storm. When the disease develops into a long-term chronic state, a potential risk might develop for malignant changes to occur in the mucosa under the effect of various cytokines. Therefore, we suggest that when following patients with COVID-19, the risk of tumorigenesis should be considered.

In summary, the ACE2-positive cells in digestive tract tissues might provide possible routes for SARS-CoV-2 infection. ACE2 expression in lung tissue increased with age, which might explain, at least partially, why older patients with COVID-19 are more likely to develop pneumonia. ACE2 expression was correlated to histological grading, suggesting that cancer patients might be more susceptible to SARS-CoV-2. Future research should focus on whether the expression of ACE2 in digestive tract organs could affect the replication of SARS-CoV-2 and whether SARS-CoV-2 infection could affect the genesis or development of tumors.

## References

[1] Coronaviridae Study Group of the International Committee on Taxonomy of Viruses. (2020). The species Severe acute respiratory syndrome-related coronavirus: classifying 2019-nCoV and naming it SARS-CoV-2. Nat. Microbiol..

[2] WHO. *Coronavirus Disease 2019 (Covid-19) Situation Report-101*. https://www.who.int/emergencies/diseases/novel-coronavirus-2019/situation-reports/ (WHO, 2020).

[3] Xu X (2020). Evolution of the novel coronavirus from the ongoing Wuhan outbreak and modeling of its spike protein for risk of human transmission. Sci. China Life Sci..

[4] He L (2006). Expression of elevated levels of pro-inflammatory cytokines in SARS-CoV-infected ACE2+ cells in SARS patients: relation to the acute lung injury and pathogenesis of SARS. J. Pathol..

[5] Li W (2007). The S proteins of human coronavirus NL63 and severe acute respiratory syndrome coronavirus bind overlapping regions of ACE2. Virology.

[6] Zhou P (2020). A pneumonia outbreak associated with a new coronavirus of probable bat origin. Nature.

[7] Moein, S. T. et al. Smell dysfunction: a biomarker for COVID-19. *Int. Forum Allergy Rhinol*. 10.1002/alr.22587 (2020). 10.1002/alr.22587 PMC7262123 32301284

[8] Mao, L. et al. Neurological Manifestations of Hospitalized Patients with COVID-19 in Wuhan, China: a retrospective case series study. *JAMA Neurol*. 10.1001/jamaneurol.2020.1127 (2020).

[9] Huang C (2020). Clinical features of patients infected with 2019 novel coronavirus in Wuhan, China. Lancet.

[10] Fang, Z. et al. Clinical characteristics of coronavirus pneumonia 2019 (COVID-19): an updated systematic review. *medRxiv*. 10.1101/2020.03.07.20032573 (2020).

[11] Zou X (2020). Single-cell RNA-seq data analysis on the receptor ACE2 expression reveals the potential risk of different human organs vulnerable to 2019-nCoV infection. Front. Med..

[12] Zhang, H. et al. The digestive system is a potential route of 2019-nCov infection: a bioinformatics analysis based on single-cell transcriptomes. *bioRxiv*. 10.1101/2020.01.30.927806 (2020).

[13] Lin, W. et al. Single-cell analysis of ACE2 expression in human kidneys and bladders reveals a potential route of 2019-nCoV infection. *bioRxiv.*10.1101/2020.02.08.939892 (2020).

[14] Xu H (2020). High expression of ACE2 receptor of 2019-nCoV on the epithelial cells of oral mucosa. Int. J. Oral. Sci..

[15] Zhang X (2015). The oral and gut microbiomes are perturbed in rheumatoid arthritis and partly normalized after treatment. Nat. Med..

[16] Whitmore SE, Lamont RJ (2014). Oral bacteria and cancer. PLoS Pathog..

[17] Chen X (2017). Poor oral health is associated with an increased risk of esophageal squamous cell carcinoma-a population-based case-control study in China. Int. J. Cancer.

[18] Maisonneuve P, Amar S, Lowenfels AB (2017). Periodontal disease, edentulism, and pancreatic cancer: a meta-analysis. Ann. Oncol..

[19] Chen W (2020). Detectable 2019-nCoV viral RNA in blood is a strong indicator for the further clinical severity. Emerg. Microbes Infect..

[20] Stuart T (2019). Comprehensive INtegration of Single-cell Data. Cell.

[21] Xu X (2020). Clinical findings in a group of patients infected with the 2019 novel coronavirus (SARS-Cov-2) outside of Wuhan, China: retrospective case series. BMJ.

[22] Zhang, J. et al. Clinical characteristics of 140 patients infected with SARS-CoV-2 in Wuhan, China. *Allergy*. 10.1111/all.142382020 (2020). 10.1111/all.14238 32077115

[23] Wang C, Horby PW, Hayden FG, Gao GF (2020). A novel coronavirus outbreak of global health concern. Lancet.

[24] Zhu, W. et al. Initial clinical features of suspected Coronavirus Disease 2019 in two emergency departments outside of Hubei, China. *J. Med. Virol*. 10.1002/jmv.25763 (2020). 10.1002/jmv.25763 PMC7228360 32167181

[25] Chen, J. et al. Individual variation of the SARS-CoV-2 receptor ACE2 gene expression and regulation. *Aging Cell.*10.1111/acel.13168 (2020). 10.1111/acel.13168 PMC7323071 32558150

[26] Liu J (2020). Longitudinal characteristics of lymphocyte responses and cytokine profiles in the peripheral blood of SARS-CoV-2 infected patients. EBioMedicine.

[27] To, K. K. et al. Consistent detection of 2019 novel coronavirus in saliva. *Clin. Infect. Dis*. 10.1093/cid/ciaa149 (2020). 10.1093/cid/ciaa149 PMC7108139 32047895

[28] Ceron, J. J. et al. Use of saliva for diagnosis and monitoring the SARS-CoV-2: a general perspective. *J. Clin. Med*. **9**, 10.3390/jcm9051491 (2020). 10.3390/jcm9051491 PMC7290439 32429101

[29] Chen, H. et al. Profiling ACE2 expression in colon tissue of healthy adults and colorectal cancer patients by single-cell transcriptome analysis. *medRxiv.*10.1101/2020.02.15.20023457 (2020).

[30] Cook KL, Metheny-Barlow LJ, Tallant EA, Gallagher PE (2010). Angiotensin-(1-7) reduces fibrosis in orthotopic breast tumors. Cancer Res..

[31] Krishnan B (2013). Angiotensin-(1-7) attenuates metastatic prostate cancer and reduces osteoclastogenesis. Prostate.

